# Characterisation of factors contributing to the performance of nonwoven fibrous matrices as substrates for adenovirus vectored vaccine stabilisation

**DOI:** 10.1038/s41598-021-00065-4

**Published:** 2021-10-22

**Authors:** Pawan Dulal, Robabeh Gharaei, Adam Berg, Adam A. Walters, Nicholas Hawkins, Tim D. W. Claridge, Katarzyna Kowal, Steven Neill, Adam J. Ritchie, Rebecca Ashfield, Adrian V. S. Hill, Giuseppe Tronci, Stephen J. Russell, Alexander D. Douglas

**Affiliations:** 1 Jenner Institute, University of Oxford, Wellcome Trust Centre for Human Genetics, Roosevelt Drive, Oxford, OX3 7BN UK; 2 Clothworkers’ Centre for Textile Materials Innovation for Healthcare, University of Leeds, Leeds, LS2 9JT UK; 3 Oxford Silk Group, ABRG, Department of Zoology, University of Oxford, Oxford, OX2 3RE UK; 4 Department of Chemistry, University of Oxford, Chemistry Research Laboratory, Mansfield Road, Oxford, OX1 3TA UK; 5 Nonwovens Innovation and Research Institute Ltd, 169 Meanwood Road, Leeds, LS7 1SR UK

**Keywords:** Vaccines, Biomaterials - vaccines

## Abstract

Adenovirus vectors offer a platform technology for vaccine development. The value of the platform has been proven during the COVID-19 pandemic. Although good stability at 2–8 °C is an advantage of the platform, non-cold-chain distribution would have substantial advantages, in particular in low-income countries. We have previously reported a novel, potentially less expensive thermostabilisation approach using a combination of simple sugars and glass micro-fibrous matrix, achieving excellent recovery of adenovirus-vectored vaccines after storage at temperatures as high as 45 °C. This matrix is, however, prone to fragmentation and so not suitable for clinical translation. Here, we report an investigation of alternative fibrous matrices which might be suitable for clinical use. A number of commercially-available matrices permitted good protein recovery, quality of sugar glass and moisture content of the dried product but did not achieve the thermostabilisation performance of the original glass fibre matrix. We therefore further investigated physical and chemical characteristics of the glass fibre matrix and its components, finding that the polyvinyl alcohol present in the glass fibre matrix assists vaccine stability. This finding enabled us to identify a potentially biocompatible matrix with encouraging performance. We discuss remaining challenges for transfer of the technology into clinical use, including reliability of process performance.

## Introduction

Vaccine immunogens are composed of complex biological macromolecules. Good immunogenicity requires stability of these molecules throughout the lifespan of a product, from production and formulation, through transportation and storage to delivery to the recipient. Extrinsic factors such as light, pH, agitation and oxidants combine with temperature fluctuations to challenge product stability. Therefore, most vaccines need to be continuously stored in refrigerators or freezers. Maintaining the cold chain and associated logistics in vaccination campaigns can contribute up to 45% of the total cost of vaccination^1^. On the other hand, damage as a result of cold chain breakages costs several million dollars annually^2^. These issues affect not only low income countries, where the cold chain is regarded as being least reliable, but also developed countries^3–5^. The vaccines responsible for eradication of smallpox and rinderpest (the only two diseases eradicated by immunisation) were both thermostable, a factor believed to contribute to their success^6^. Development of technologies to enhance vaccine thermostability has therefore been a major focus of research effort^7^.

Viral vectored vaccines, in particular adenoviruses, are versatile platforms for development of novel vaccines against emerging pathogens^8,9^, malaria^10,11^, HIV-AIDS^12,13^, tuberculosis^14^ and influenza^15,16^. The platform has proven its value in response to the COVID-19 pandemic, providing the basis for vaccines developed by Janssen, the Gamaleya Institute and Cansino, in addition to the Oxford/Astrazeneca vaccine (Vaxzevria) which we contributed to developing. The rapid platform manufacturing process is attractive for emergency response^17,18^. Successful thermostabilisation of adenovirus vectored vaccines could thus be valuable for products targeting a wide range of diseases.

There have been a number of efforts to enhance stability of adenovirus-based vaccines in liquid formulations, achieving stability for several months at 2–8 °C or a number of weeks at 15–30 °C^19–21^. Other groups have reported improvement in stability by drying vaccines using lyophilisation^22,23^, spray drying^24–26^, and nano-patch technology^27^, in some cases demonstrating short-term stability (weeks) at temperatures up to 37 °C^20^.

We have previously reported that drying a formulation of adenovirus in glass forming sugars upon a fibrous matrix such as paper or a wetlaid nonwoven fabric permitted full recovery of viable and immunogenic virus, even after 6 months at 45 °C^28–30^. We are not aware of any other reports of adenovirus stabilisation at such temperatures. We refer to this method as sugar-matrix thermostabilisation (SMT). As well as thermostabilisation performance, advantages of SMT include a relatively short process duration (in some cases as little as 18 h) and avoidance of the stress of extreme in-process temperature exposure involved in lyophilisation and spray drying.

Our previous published SMT work used a glass fibre matrix and a polypropylene matrix; we reported that the glass fibre matrix achieved better stability than the polypropylene matrix^28^. Glass fibre matrix is however prone to shedding of non-biocompatible fibres, and so not suitable for clinical translation.

**Figure 1:**
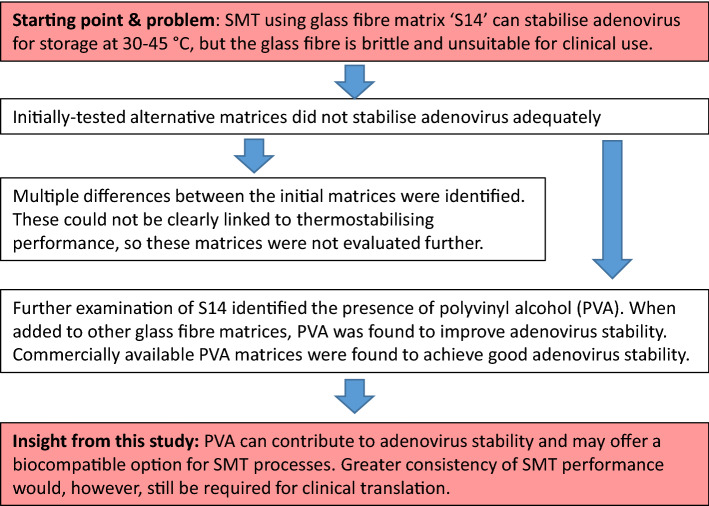
Scheme of investigation. The research strategy used in the current work is presented in terms of research questions and answers, leading from a starting point and problem statement to a conclusion and identification of a key remaining challenge.

Here, we present results from an effort to characterise the factors contributing to the performance of glass fibre matrix and to test alternative matrices more suitable for clinical use. An overview of our strategy is presented in schematic form in Fig. 1.

## Results

### Glass fibre matrix achieves good stability but is not suitable for clinical development

**Figure 2:**
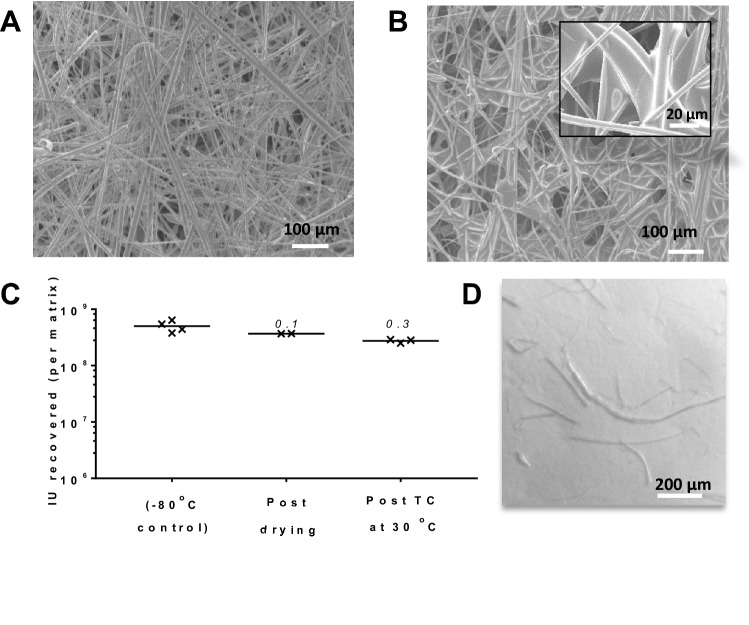
SMT on S14 results in thermostable vaccine embedded in sugar glass. (**A**) and (**B**) show scanning electron micrographs of S14, respectively before and after loading and drying of 0.5 M (80:20) trehalose:sucrose. In panel (**A**), the arrangement of fibres in S14 is apparent (× 200 magnification, 100 µm scale bar). In panel (**B**), sugar glass films are visible between fibres (main panel at × 200 magnification with 100 µm scale bar, inset at × 1000 magnification with 20 µm scale bar). Panel (**C**) shows recovery of vaccine from matrix loaded with a human dose of adenovirus, both immediately post-drying and after thermochallenge at 30 °C for 1 month. Results plotted illustrate total virus recovery from each replicate matrix (n = 4 for − 80 °C comparator, n = 2 for post-drying, n = 3 for post-thermochallenge), with lines indicating the mean of the replicates. Numbers above each result show log_10_-fold loss from the − 80 °C control. Panel (**D**) shows a representative light microscopy image of fibres shed from S14 after vaccine reconstitution (scale bar 200 µm).

In our previously published work, we had used low vaccine doses (1.1 × 10^10^ viral particles per matrix, as compared to a typical human dose of 5 × 10^10^ viral particles), applied to the glass fibre matrix ‘Standard 14’ (S14, GE Healthcare, Fig. 2A)^28^. We speculate that the fibrous matrix provides a high surface area, favouring relatively rapid drying of the product despite the gentle conditions used (18–24 h at 20–23 °C and atmospheric pressure). The process results in films of vitreous ‘sugar glass’ embedded in the matrix (Fig. 2B). As previously reported, this ‘base-case’ process achieves a sugar glass transition temperature (T_g_) after drying of 47–55 °C, with moisture content 3–5% of total solute dry weight^28^.

We have now dried full human doses (5 × 10^10^ virus particles, approximately 5 × 10^8^ infectious units [IU]) of a simian adenovirus vectored rabies vaccine (ChAdOx2 RabG) upon 1 cm^2^ of the matrix, with < 0.1 log_10_-fold loss in-process (i.e. during desiccation) and approximately 0.3 log_10_-fold loss after thermochallenge at 30 °C for a month (Fig. 2C)^30^. For explanation of the log_10_-fold loss metric, please see “Methods”.

Despite the good thermostabilisation performance of the glass fibre matrix, we were concerned that the brittleness of glass fibre would result in shedding of fibres during the process of vaccine reconstitution. Indeed, macroscopic damage to the matrix was sometimes apparent at the point of reconstitution. We therefore sought to quantify subvisible particles (0.1–100 μm) in the reconstituted vaccine. Application of a pharmacopoeial light microscopy method revealed numerous glass fibres (Fig. 2D). Although passing the reconstituted solution through a 5 µm filter needle removed virtually all detectable glass particles, reducing the particulate burden within pharmacopoeial limits, such a method is unsuitable for clinical development, with the possible exception of very early-phase studies^31^.

### Initial selection of commercially-available matrices for evaluation

In our previously published work, we reported that the S14 matrix achieved better vaccine thermostability than an alternative commercially-available polypropylene-based matrix (HDC^®^II J200, Pall Corporation). We therefore sought to identify alternative commercially-available matrices which might offer thermostability equivalent to or better than that achieved with S14, but without the problem of shedding of non-biocompatible fibres. A set of seven matrices were selected based on manufacturers’ product specifications claiming low fibre shedding, low chemical leaching and compatibility with sterilisation either by dry heat, steam or gamma-radiation sterilisation (Table 1). Henceforth matrices are referred to, for clarity of identification, in terms of their fibre material and the manufacturer’s product name.

**Table 1:** Basic characteristics of studied matrices.

Matrix name (manufacturer product reference)	Manufacturer (sub-brand if applicable)	Main material	PS20 treatment?	Absorption > 20 µL/cm^2^ H_2_O	Loading capacity (µL/cm^2^)	Fibre diameter (µm) ± SEM	Mean pore size (µm)	Thermostabilisation performance (log_10_-fold infectivity loss)
S 14	GE (Whatman)	Glass fibre	×	✓	55	4.2 ± 0.3	20	0.3
J200	Pall	Polypropylene	✓	✓	36	21.7 ± 0.4	20	1
23100	Hollingsworth- Vose	Polyester	✓	✓	43	4.4 ± 0.4	11	1.3
33100L	Hollingsworth- Vose	Polyamide	✓	✓	36	4.8 ± 0.4	27	0.9
Cyclopore (PC)	GE (Whatman)	Polycarbonate	✓	×	Excl.	Excl.	Excl.	Excl.
31 ET CHR	GE (Whatman)	Cellulose	×	✓	40	15.4 ± 1.0	1.2	2.4
Leukosorb (Leu)	Pall	Polyester^a^	×	✓	40	3.5 ± 0.2	8	1.7
Conjugate pad (CP)	Pall	Glass fibre^b^	×	✓	30	12.3 ± 0.4	N/A	0.9
Asymmetric polysulfone (AS)	Pall	Polysulfone	✓	×	Excl.	Excl.	Excl.	Excl.

Loading capacity was measured by weight measurement of matrices before and after full impregnation in water. Fibre diameter was measured using Image J from SEM micrograph images. Mean pore size are as provided in suppliers’ specification (in one case N/A denotes data not available). Thermostabilisation performance presented is loss of infectivity after vaccine drying and thermochallenge for 1 week at 45 °C. Excl. denotes matrices excluded from further analysis on basis of loading capacity.

^a^Proprietary composition, but macroscopic, microscopic and FTIR spectrum appearances are consistent with polyester (data not shown); this matrix is henceforth referred to as polyester.

^b^Henceforth referred to as ‘glass fibre (conjugate pad)’ for avoidance of confusion with S14.

We initially screened all matrices for suitable loading capacity (> 20 µL of deionised water/cm^2^). Matrices which did not absorb 20 µL/cm^2^ (without visible remaining beads of water within 2 s) were re-tested after treatment with 2% w/v polysorbate 20 solution. Matrices which did not absorb 20 µL/cm^2^ after detergent treatment were not studied further. We proceeded to further study of the remaining five matrices along with the two previously tested matrices (glass fibre S14 and polypropylene J200).

**Figure 3:**
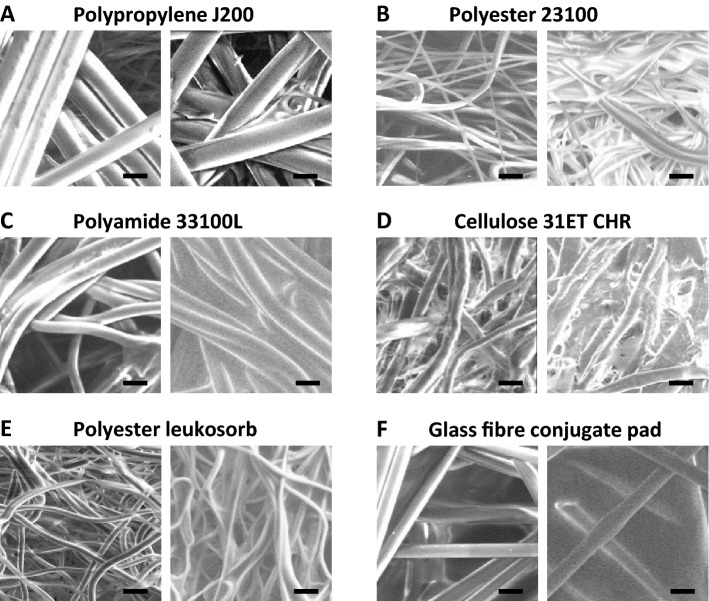
Physical appearance of fibres and sugar glass formed in matrices. Scanning electron microscopy images of six different matrices at × 1000 magnification. Within each panel, the left-hand image shows empty matrices and the right-hand image shows fibres after loading and drying of 0.5 M (80:20) trehalose:sucrose solution. Scale bar shows 20 µm in each image.

The architecture of the selected matrices was characterised by scanning electron microscopy (Fig. 3). Fibre diameters estimated for each matrix type using Image J analysis are presented in Table 1.

Fibres in the polypropylene (J200) matrix (Fig. 3A) and glass fibre (conjugate pad) matrix (Fig. 3F) shared the straight, rod-like fibre morphology seen in the original glass fibre (S14) matrix (Fig. 2A,B) but had larger fibre diameters of 10–20 μm (as compared to 4 μm in the S14 glass fibre sample). The remaining four matrices (Fig. 3B–E) exhibited a greater degree of curl along their length. A film was apparent on the glass fibre matrix (conjugate pad) (Fig. 3F). This resembled a binder (a glue-like substance used during the production of non-woven matrices, to hold the fibres together and ensure mechanical strength of the product). Matrices loaded with 0.5 M sugar solution and dried at room temperature for 24 h also showed differences in the distribution of the sugar glass intercalated between the fibres (Fig. 3). Distinct films of sugar glass between the fibres were visible on the two glass fibre matrices, but not in the polyamide (33100L), polyester (leukosorb) or polypropylene (J200) matrices. The sugar loaded polyester (23100) matrix and cellulose (31 ET CHR) matrices showed a glazing effect on the fibres, with discrete sugar glass films being apparent.Adenovirus vaccine vectors were formulated in 0.5 M TS and dried on the five selected matrices, with glass fibre (S14) and polypropylene (J200) matrices as comparators of known performance. Dried matrices were thermochallenged for a week at 45 °C prior to reconstitution and infectivity titration. Marked thermostabilisation performance differences between the matrices were apparent (Table 1), with none of the alternative matrices matching the performance of S14. The next-best-performing matrices were polyamide (33100L) and glass fibre (conjugate pad), with infectivity loss of 0.9 log_10_-fold in each. The cellulose-based matrix (GE) performed most poorly.

To understand the highly variable thermostabilisation performance of the matrices, we investigated whether performance could be correlated with the physical properties of the matrices or the sugar glass formed. 0.5 M [50:50] trehalose sucrose was loaded into each matrix and dried at room temperature and < 5% relative humidity. Dried samples were then subjected to modulated differential scanning calorimetry (DSC) to measure glass transition temperature (Tg) onset temperature and enthalpic recovery of the sugar glass formed on the matrices. The glass transition temperature (T_g_) indicates the temperature at which a low mobility sugar glass changes to a highly mobile rubbery state and is known to be related to product stability in dry formulations^32^. Enthalpic recovery is a measure of energy dissipated as a glass progresses through equilibrium and can reflect molecular rearrangement during storage or physical ageing^33^.

**Figure 4:**
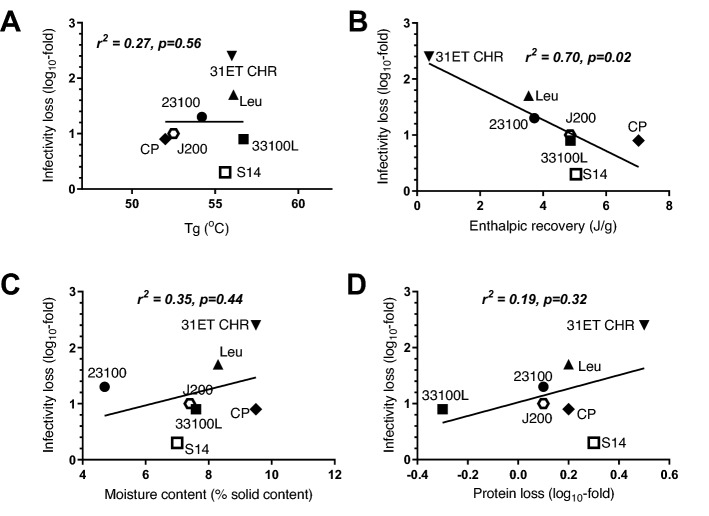
Relationships of sugar glass properties with vaccine thermostability on various matrices. Each panel relates the thermostabilisation performance of the various matrices (after 1-week thermochallenge at 45 °C, Y-axis, data shown in Table 1) to a potentially explanatory variable on the X-axis: glass transition temperature (panel **A**); enthalpic recovery (panel **B**); moisture content (panel **C**); and protein recovery (panel **D**). Results of Pearson correlation analysis are shown within each panel. DSC measurements (panels **A** and **B**) were made in singlicate, Karl–Fischer measurements (panel **C**) were made in duplicate, and protein loss measurements (panel **D**) were made in triplicate. For panels (**C**) and (**D**), points represent the mean measurement.

The T_g_ was similar for all matrices, observed over a narrow range between 52 and 56 °C, and did not correlate with thermostability (Fig. 4A). A possible correlation was observed between thermostabilisation performance and high enthalpic recovery (Fig. 4B, r^2^ = − 0.70, p = 0.02), as seen with glass fibre matrices conjugate pad and S14 followed by polypropylene (J200) based matrix; to our knowledge this is not a parameter amenable to controlled manipulation and we did not evaluate this further. There was substantial variation in the residual moisture content of products dried under the same conditions on different matrices (Fig. 4C), the best performing matrix, S14 having relatively low residual moisture. Recovery of a model protein (lysozyme) after desiccation and reconstitution did not predict thermostabilisation performance (Fig. 4D).

Given the multiple parameters differing between the matrices, and the lack of a readily modified parameter correlated with performance, this line of enquiry was not pursued further.

### Characterisation of S14 glass fibre matrix

Given our inability to identify a commercially-available matrix suitable for clinical application, we turned our attention to detailed characterisation of the best-performing S14 matrix, with a view to future production of a similar matrix from biocompatible materials.

We initially performed a more extensive characterisation of the physical properties of the matrix, with results as shown in Table 2.

**Table 2:** Physical characteristics of glass fibre (S14) and PVA matrices.

	S14	PVA1	PVA2	PVA3	PVA4	PVA5	PVA6
Areal density (g·m^−2^)	55	12	24	36	48	60	84
Thickness (μm)	510	40	73	136	170	190	270
Fibre diameter (µm)	3.4	21	21	22	23	22	22
Fibre length (mm)	4.4	4.4	4.2	4.1	4.5	4.0	4.3
Largest pore diameter (µm)	133	ND	ND	ND	ND	ND	ND
Mean flow pore diameter (µm)	22	ND	ND	ND	ND	ND	ND
Smallest pore diameter (µm)	6	ND	ND	ND	ND	ND	ND
Porosity (%)	95	75	72	78	76	73	73
Capillary constant (cm^5^)	7.7 × 10^–7^	ND	ND	ND	ND	ND	ND
Virus-aqueous sugar solution contact angle (°)	31	ND	ND	ND	ND	ND	ND
Virus-aqueous sugar solution surface tension (mN·m^−1^)	64	ND	ND	ND	ND	ND	ND

*ND* not done. Characteristics of S14 are relevant throughout the “Results” section. PVA matrices PVA1-6 are introduced in the final subsection of “Results”, and are relevant only to that subsection. The nomenclature ‘PVA1-6’ refers to six commercially available PVA matrices from within a range produced by Kuraray Co (Japan), as described in the text.

Use of chemical binders is common in the production of nonwoven fabrics such as S14 to provide strength and desirable surface properties. In view of the possibility that a binder might be contributing to S14’s thermostabilisation performance, we investigated whether such a binder could be identified in the matrix.

**Figure 5:**
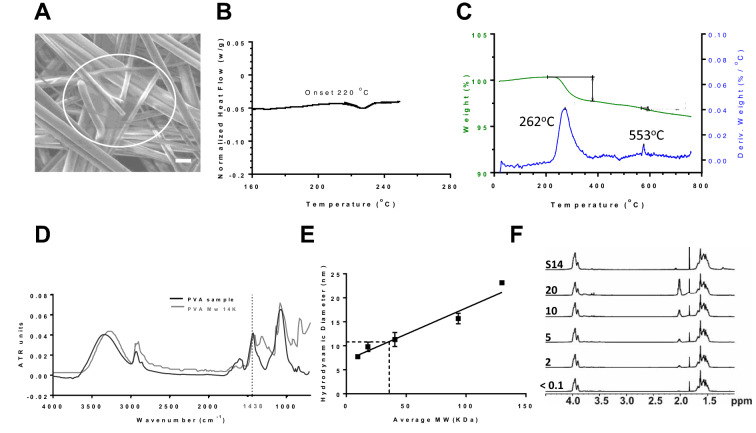
Chemical properties of glass fibre matrix. Panel (**A**) shows a scanning electron microscopy image indicating presence of binder in glass fibre (S14), visible as a film in the white-circled area. Scale bar shows 10 µm. Such areas were relatively sparse, compared to the extensive films apparent in matrices imaged after loading and drying of trehalose:sucrose solution (Fig. 2B). Panel (**B**) shows a differential scanning calorimetry thermogram of glass fibre (S14), representative of duplicate measurements. The graphs show heat flow as a function of temperature during scanning from 40 to 250 °C. Panel (**C**) displays thermogravimetric analysis of glass fibre (S14), showing a thermal decomposition step. Weight losses (green lines) and the rate of weight loss (i.e. derivative, %/°C) (blue lines) are shown. Panel (**D**) shows the sample spectrum obtained from attenuated total reflectance Fourier transform spectroscopy (ATR-FTIR) for the binder recovered from glass fibre (S14) (black), and a reference library spectrum for PVA (grey). Panel (**E**) shows estimation of the molecular weight of the binder extracted from glass fibre (S14) by DLS. Points and solid line show a standard curve generated using PVA of known MW. Dashed lines indicate the hydrodynamic radius of the PVA extracted from S14 (11 nm) and the inferred MW (36 kDa). Points and error bars indicate the mean and range respectively of duplicate DLS measurements. Panel (**F**) shows 1H NMR spectra used to estimate the percentage hydrolysis of the binder extracted from glass fibre (S14). The upper spectrum (labelled S14) is that of the extract, with unknown percentage hydrolysis and hence an unknown percentage of monomers bearing acetyl groups. The five spectra below were obtained using standards prepared by proportionately mixing 80% and 100% hydrolysed PVA to achieve a range of acetyl group content ranging from < 0.1% up to 20% (as per labels to left of panel). The X-axis indicates chemical shift measured in parts per million (ppm), with relative intensity indicated in the Y-axis dimension i.e. vertically.

Scanning electron microscopy demonstrated film-like material which could represent binder covering fibres in some sections of the S14 sample (Fig. 5A). Differential scanning calorimetry and thermogravimetric analysis (Fig. 5B,C) demonstrated the presence of a material with a melting point of 220 °C and a degradation temperature at 260 °C.

For further characterisation, the binder was extracted in liquid and freeze dried. Fourier transform infrared (FTIR) spectroscopy of the extract provided a fingerprint spectrum, which matched closely with the expected spectrum of polyvinyl alcohol (PVA) (Fig. 5D)^34,35^. The presence of PVA in the matrix is also supported by above DSC results, indicating a melting transition in the range of 180–240 °C, typical of PVA^36^.

The solubility and other properties of PVA vary widely according to molecular weight (MW) and degree of hydrolysis, and so we sought to further characterise the presumed PVA extracted from S14. We used dynamic light scattering (DLS) to compare the hydrodynamic radius of the PVA extract to those of PVA samples of known MW. The results were consistent with a MW in the range of 35 kDa (Fig. 5E).

We then used 1H NMR to estimate the degree of hydrolysis of the polymer. The 1H NMR spectrum obtained (Fig. 5F) was consistent with PVA, with a 2:1 ratio of the areas under the peaks at ~ 1.6 ppm and 3.9 ppm (corresponding to hydrogens in the CH_2_ and CH environments respectively). Results obtained using a range of standards of varying percentage hydrolysis showed a clear relationship of the size of a peak at 2.05 ppm to the acetyl group content (the presence of which, in a sample of PVA, indicates incomplete hydrolysis). The spectra of the completely hydrolysed standard and the S14 extract were similar, with only a trace of a peak in this area, and so we concluded that the PVA extracted from S14 is likely to be completely hydrolysed.

### PVA enhances adenovirus stabilisation by SMT

Having identified PVA in the best-performing matrix, S14, we investigated whether the PVA may function not only as a binder but might actually contribute to vaccine thermostabilisation.

**Figure 6:**
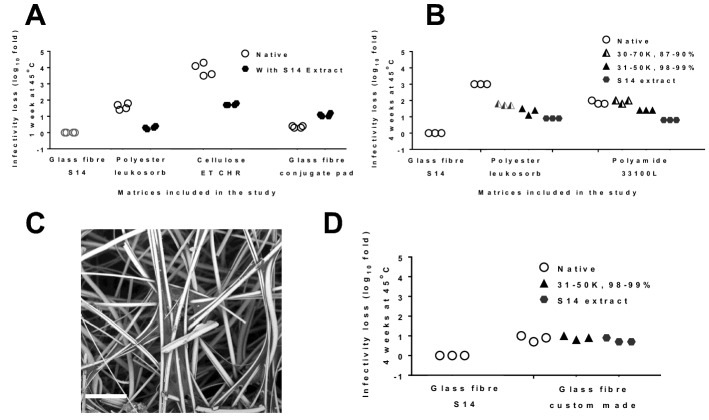
PVA improves vaccine thermostability on matrices. A PVA-containing extract prepared from S14 was dried onto various fresh matrices, followed by drying of vaccine in 0.5 M (50:50) trehalose-sucrose onto the treated matrices and untreated comparator matrices. Viable virus recovery was assessed after thermochallenge as indicated for each panel. S14 was included as a comparator in each experiment. Panel (**A**) shows loss in infectivity titre of vaccine in the absence (open circles) or presence (solid circles) of PVA extracted from glass fibre (S14), as compared to − 80 °C stored liquid control, after 1 week at 45 °C. Points indicate individual samples (four replicates per condition). Panel (**B**) shows difference in loss in infectivity titre of vaccine loaded in polyester (leukosorb) and polyamide (33100L) matrix modified by deposition of three different types of PVA prior to application and drying of vaccine. Dried samples were subjected to thermochallenge for 4 weeks at 45 °C. Points indicate individual samples (three replicates per condition). Panel (**C**) shows a scanning electron microscope image of the custom-made glass fibre matrix. Scale bar shows 50 µm. Panel (**D**) compares loss in infectivity titre of vaccine post-thermochallenge between standard 14 and custom-made glass fibre matrix modified by deposition of two different types of PVA prior to application and drying of vaccine. Dried samples were subjected to thermochallenge for 4 weeks at 45 °C. Points indicate individual samples (three replicates per condition).

We reasoned that treatment with PVA might enhance the thermostabilisation performance of relatively poorly-thermostabilising matrices. We observed that application of PVA extracted from glass fibre (S14) significantly improved thermostabilisation performance of two of the three tested matrices, as assessed by infectious virus recovery after a 4 week 45 °C thermochallenge. Recovery from the polyester matrix (Leukosorb) was enhanced by 1.3 log_10_-fold, while enhancement from the cellulose matrix (ET CHR) was enhanced by 2 log_10_-fold (Fig. 6A). Addition of PVA to a glass fibre matrix (Pall’s conjugate pad) was not beneficial. This matrix already contains a binder (Fig. 3F), possibly PVA.

We proceeded to test whether the degree of hydrolysis of PVA had any impact on vaccine thermostability at 45 °C for an extended period of 28 days. We tested polyester (Leukosorb), polyamide (33100L) and a custom-made glass fibre-based matrix after treatment with PVA extracted from S14 and two other commercially sourced polymers (Sigma) which were similar in size but differed in degree of hydrolysis (30–70 kDa/87–90% hydrolysed, and 31–50 kDa/> 99% hydrolysed). We again observed substantial improvements in thermostabilisation performance, exceeding a 1 log_10_-fold increase in infectious virus recovery after thermochallenge, when PVA was applied to matrices which did not contain PVA at baseline (polyamide and polyester) (Fig. 6B). The greatest enhancement was seen with the S14 extract, followed by the fully hydrolysed PVA, with the least enhancement seen with the incompletely hydrolysed PVA (Fig. 6B).

Next, we tested whether the understanding we had gained would enable us to produce an ‘in-house’ wet-laid glass fibre matrix which could replicate S14’s thermostabilisation performance. The new matrix had structural properties similar to those of S14 (area density 50.6 ± 1.5 g·m^−2^, thickness 0.56 ± 0.02 mm, mean flow pores 25 ± 2.3 µm, absorption capacity 11.1 ± 0.3 g/g, composed of borosilicate glass fibres with diameter 3.6 ± 1.8 and length 1.3 ± 0.6 mm; see Table 2 for data relating to S14), and a similar appearance (Fig. 6C). Although thermostabilisation performance of the custom-made matrix did not exactly match that of S14 (Fig. 6D), it was closer than had previously been achieved with any of the other tested matrices (Table 1 and Fig. 6B). As expected, further modification of the matrix with additional PVA had no effect.

### Matrices composed of PVA have potential to achieve good stability with reduced shedding

Having established that the presence of PVA in glass fibre matrices could enhance adenovirus stability, we identified six commercially-available matrices composed entirely of PVA (both fibres and binder). The only identifiers provided by the supplier (Kuraray Co, Japan) for these matrices were the catalogue numbers BFN1-6. Henceforth these matrices are referred to as PVA1-6. Physical characteristics of these matrices were measured (Table 2). The PVA matrices had a range of thicknesses and hence areal densities, but otherwise shared similar structures as each other. Compared to S14, the PVA matrices had porosity somewhat lower than that of S14 (75% vs 95%), substantially thicker fibers than S14 (c. 22 μm vs 3.4 μm), and similar fiber length (c. 4 mm).

**Figure 7:**
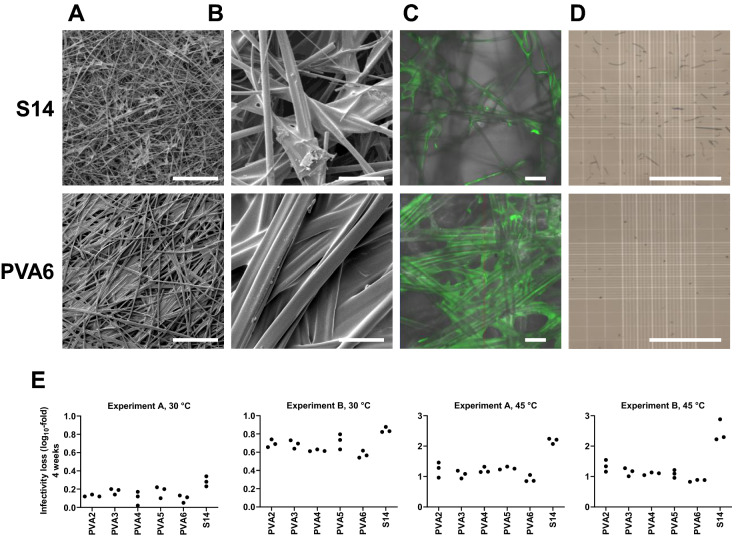
Characterisation of PVA matrices. Panels (**A**)–(**D**) compare S14 (upper row of images) and PVA6 (lower row). Images with matrices PVA2-5 were similar to those shown for PVA6. Panels (**A**)–(**C**) show the distribution of sugar-glass film after drying of 0.5 M (50:50) trehalose:sucrose solution (50 μL/cm^2^): (**A**) shows SEM at low magnification (scale bar = 500 μm); (**B**) shows SEM at higher magnification (scale bar = 50 μm); (**C**) shows confocal laser scanning microscopy of the distribution of fluorescein-labelled lysozyme within the film (scale bar = 50 μm). (**D**) Show results of light microscopy for subvisible particle detection after reconstitution of dried 0.5 M (50:50) trehalose:sucrose solution (scale bar = 1 mm). (**E**) compares loss in infectivity titre of vaccine post-thermochallenge between PVA-based matrices (PVA2-6) and S14 glass fibre matrix. As indicated in the graph headings, dried samples were subjected to thermochallenge for 4 weeks at 30 or 45 °C, and the experiment was performed in duplicate. Points indicate individual samples (three replicates per condition).

Matrices were then screened for loading capacity. PVA2-6 had loading capacity > 50 μL/cm^2^; a visible bead of fluid remained on PVA1 at 50 μL/cm^2^ and it was therefore excluded from further investigation. We then imaged matrices after drying of mock samples. Matrices were subjected to SEM after drying of 50 μL/cm^2^ 0.5 M trehalose:sucrose (50:50) (Fig. 7A,B), and to confocal laser scanning microscopy after drying of a similar sample with added 10 mg/mL fluorescein-labelled lysozyme as a model proteinaceous load (Nanocs Inc, USA) (Fig. 7C). As compared to S14, it was apparent that drying on PVA matrices resulted in more widespread formation of sugar film around and between the fibres. We next assessed the mechanical robustness of the PVA matrices: upon reconstitution of a dried sugar solution, there was little evidence of fibre shedding (Fig. 7D).

Finally, we tested the performance of the PVA matrices in stabilisation of adenovirus. When assessed by thermal challenge at 30 °C or 45 °C for 1 month, vaccine stabilisation on PVA2-6 was consistently favourably comparable to that on S14 (Fig. 7E). We did however observe substantial variation between the duplicate experiments in stability at 30 °C, and stability at 45 °C on all matrices (including S14) was inferior to that seen previously.

## Discussion

The starting point for the present study was the observation, in our previous work, that the non-biocompatible glass-based S14 matrix out-performed a polypropylene matrix^28^. This posed the question of which properties of the matrix could be relevant for SMT performance, and whether a more suitable matrix than S14 could be identified for clinical translation.

Our initial attempts to find a suitable commercially available matrix yielded disappointing results. Adenovirus stability on the tested matrices was poor (Table 1). Multiple variables differed between each matrix, and we were therefore unable to perform experiments to clearly isolate the effect of a single variable. There was not a clear relationship between characteristics of the matrices and their stabilisation performance (Figs. 3 and 4), with the possible exception of high enthalpic recovery of sugar glass (which is not a parameter which can readily be ‘designed in’ to a new matrix).

Our ability to use analysis of commercially available matrices to draw conclusions to guide design of new biocompatible matrices was thus limited. We therefore changed our strategy, seeking to characterise S14 in detail and produce similar matrices ‘in-house’, allowing us to identify features of S14 contributing to its stabilising performance.

Most significantly we found that PVA, present on the S14 matrix as a binder, appears to contribute to the stability of adenovirus (Figs. 5 and 6). It was shown that fully hydrolysed PVA, similar to that we extracted from S14, was most beneficial in thermostabilisation (Fig. 6). Polyvinyl alcohol is potentially suitable for use as an excipient in vaccine formulations: it is ‘generally regarded as safe’ (GRAS) and is also a FDA approved inactive ingredient for parenteral use^37^. PVA has previously been explored as an excipient in a number of studies of bio-macromolecular stability. It has been found to be beneficial in some formulations of proteins, including insulin^38^, but benefit has not been seen consistently in other studies^39–41^. PVA may contribute to protein stability by hydrogen bonding of hydroxyl groups in PVA to the proteins. In dried protein formulations, PVA has also been shown to prevent deamidation more potently than another widely used polymeric excipient, polyvinyl pyrrolidone^42^.

We were subsequently able to demonstrate that matrices composed entirely of PVA could achieve stability performance favourably comparable to S14. PVA is biocompatible and these matrices shed few fibres during sample reconstitution. These matrices therefore appear more suitable for clinical application than our previous gold-standard glass-fibre matrix.

This work was performed as part of an effort to develop the SMT technology for Good Manufacturing Practice (GMP) compliant manufacture of a product suitable for an early-phase clinical trial. This work faced two principal challenges: identification of a suitable matrix, and identification of means of performing the process reliably under GMP-compliant conditions. We believe PVA matrices offer a potential solution to the former, but we have not yet been able to achieve sufficient process reliability to support clinical translation.

Figure 7E shows an example of process variability between replicate experiments, and overall poor performance in terms of stability at 45 °C. These experiments were performed by drying in a lyophilizer (with atmospheric pressure and room temperature, i.e. using the lyophilizer only to provide a low humidity temperature-controlled environment). We have however observed similar process variability across numerous additional experiments drying within a glovebox (as used for experiments 1–5) or dessicator. Extensive efforts to find optimal conditions (investigating the effects of changing temperature and duration of drying, as well as excipient composition) have been unsuccessful in resolving the variability.

We hope that future work may resolve these remaining challenges. If consistency of performance could be achieved, SMT offers ability to stabilise adenovirus for months at 45 °C which is, to our knowledge, unmatched by other stabilising approaches. The current study, however, highlights the potentially beneficial effect of PVA upon adenovirus stability. This may be of relevance not only for the SMT approach, but potentially for other approaches to stabilising vaccines based upon this important platform.

## Methods

### Viruses and infectivity titration

Simian adeno virus vectors ChAd63-METRAP and ChAdOx2-RabGP were prepared, purified and tested for quality by the Jenner Institute Viral Vector Core Facility, as previously described^30,43^. Viruses were dialysed against either a previously used storage buffer (10 mM Tris, 7.5% w/v sucrose, pH 7.8) or unbuffered 0.5 M (50:50) trehalose and sucrose and stored at − 80 °C as stock. ChAdOx1 expressing *Photinus pyralis* luciferase^44^ was prepared as previously described, with tangential flow filtration into unbuffered 0.5 M (50:50) trehalose and sucrose^45^. Typical preparations were supplied and stored at a titre of c. 1 × 10^12^ virus particles (VP) per mL, corresponding to c. 1 × 10^10^ infectious units (IU) per mL and hence a particle: infectivity ratio of c. 100.

For infectivity titration of ChAd63-METRAP and ChAdOx2-RabG, duplicate fivefold serial dilutions were prepared in complete DMEM (10% FCS, 100 U penicillin, 0.1 mg streptomycin/mL, 4 mM l-glutamine) and used to infect 80–100% confluent HEK293-TRex cells (ThermoFisher) grown in 96-well plates (BD Purecoat Amine, BD Biosciences, Europe). Infected cells were immunostained and imaged as previously described^46^. Wells containing 20–200 spots were used to back-calculate recovered infectious units.

Infectivity titration of ChAdOx1 luciferase was performed as above with the exception that, 24 h after infection, cells were lysed by addition of 1% Triton X-100 (Sigma) and luciferase activity measured using a Bright-Glo assay kit (Promega). Infectivity was calculated by interpolation on a standard curve prepared by serial dilution of a sample of known infectivity.

### Drying, thermochallenge and reconstitution

Stock vaccines were thawed and diluted into unbuffered 0.5 M trehalose–sucrose solution. Dilution factors, final viral titres, and the trehalose:sucrose ratio varied between experiments, as stated in figure legends.

For data other than Fig. 7E, fibrous matrix was cut into 100 mm^2^ pieces, loaded with vaccine or sugar solutions (as described in individual figure legends) and transferred to a glove box (Coy Laboratory Products). An activated silica bed within the chamber and circulation of air through desiccation capsules containing anhydrous calcium sulphate (Drierite™, W.A. Hammond Drierite Co.), regulated by a humidity controller (Series 5000, Electro-tech Systems), was used to maintain relative humidity beneath 5%. A portable datalogger (AET-175, ATP instruments) was used for recording changes in relative humidity and temperature during the desiccation process. The temperature inside the enclosed glove box remained between 22 and 25 °C for all experiments.

For data shown in Fig. 7E, drying was performed similarly except for use of a lyophilizer rather than glovebox to provide a room temperature, atmospheric pressure low humidity environment: humidity was lowered by setting the condenser to − 70 °C, while the sample temperature was controlled by setting the shelf temperature to 20 °C.

Samples were transferred into 2 mL glass vials, stoppered and crimp sealed under dry conditions within the glove box prior to further use. Samples undergoing thermochallenge i.e. storage at elevated temperature (typically 45 °C for 1 or 4 weeks) were stored within secondary packaging (moisture barrier bags).

For experiments involving reconstitution of dried samples, this was performed by addition of phosphate buffered saline (Sigma), followed by brief vortexing of the vial (1 ± 0.5 s, three times). Virus infectivity after reconstitution was assayed as described above. Recovery of infectious virus was quantified by comparison to a control sample of the starting material, included on the same assay plate. Between the set-up of an experiment and the assay of recovered infectivity, control samples were stored at − 80 °C in aqueous buffer (under which conditions loss of infectivity is known to be negligible).

Recovery was calculated in terms of log_10_-fold loss in the total infectious virus content of the matrix i.e. log_10_-fold loss = log_10_(infectious units dried on matrix based on − 80 stored sample) − log_10_(infectious units recovered from matrix). 0.3 log_10_-fold loss thus implies c. 50% recovery, 0.5 log_10_-fold loss implies c. 30% recovery, and 1 log10-fold loss implies 10% recovery.

### Karl Fischer moisture analysis

Residual moisture content in single matrix post-desiccation was determined with a Karl Fischer moisture analyser equipped with coulometer (Metrohm), and Hydranal-Coulomat titration solution (Honeywell, Fluka) in accordance with the manufacturers’ recommendations. A standard was used to calibrate the instrument performance (lactose standard 5%, MerckMillipore). Residual moisture content was expressed as a percentage (calculated from the measured mass of water as a proportion of the calculated mass of solutes in the loaded sample).

### Measurement of subvisible particles

To produce the data shown in Fig. 2D, 100 mm^2^ pieces of glass fibre (GE Whatman Standard 14 [henceforth S14]) were cut, autoclaved at (121 °C, 15 min) and loaded with 50 µL of sugar solution prior to drying in the glove box, vialling and reconstitution as described above. Reconstituted solution from 10 vials was aspirated using a syringe with a conventional 20G needle (BD Biosciences), to produce a single pooled unfiltered sample. Reconstituted solution from a further 10 vials was aspirated using a 5-μm filter needle (BD Biosciences), to produce a single pooled filtered sample. Both pools were diluted to a final volume of 25 mL and tested for sub-visible particles by light microscopy according to Ph.Eur (2.9.19)/USP $\left\langle {788} \right\rangle$ by a commercial testing laboratory (Reading Scientific Services Limited).

The data shown in Fig. 7D was collected using a similar but non-pharmacopoieal in-house approach, in which unfiltered solution from reconstitution of samples dried on S14 and PVA matrices was subjected to light microscopy (Leica M205C).

### Scanning electron microscopy

To produce the images shown in Figs. 2, 3, 4, 5, 6, untreated matrices (i.e. without sputter coating) were loaded onto aluminium mounts using carbon conductive tabs and imaged using a Zeiss-Evo LS15 variable pressure scanning electron microscope (SEM) equipped with variable pressure secondary electron detector (Carl Zeiss Ltd). Imaging was performed at a chamber pressure of 50 Pa air and accelerating voltage of 15 kV.

To produce the images shown in Fig. 7A,B, samples were sputter coated with gold with the coating thickness of 30 nm using a Quorum Q150RS coating unit. They were then imaged using a Jeol JSM-6610LV scanning electron microscope with 5 kV accelerating voltage and beam spot size at setting 40.

Further analyses of images such as measurement of fibre diameter were made using Image J software (freely available, National Institutes of Health). At least 40 measurements per matrix were performed, using images taken at varying magnification.

### Confocal laser scanning microscopy

10 mg/mL Fluorescein-labelled lysozyme (Nanocs Inc, USA) was spiked into the 0.5 M trehalose:sucrose (50:50) solution and dried as above before examination by fluorescence confocal microscopy (DMI6000 B, Leica Microsystems, Germany).

### Differential scanning calorimetry

The glass transition temperature (T_g_) of sugar glass in matrices was measured immediately after drying had completed using Differential Scanning Calorimetry (DSC). The melting point of the binder in untreated glass fibre (S14) was measured using DSC (Q2000, TA instruments). The instrument was purged with dry nitrogen (50 mg/mL) continuously during sample measurement. Calibration was performed prior to measurements using a certified reference material (Indium) for temperature and heat flow accuracy.

Multiple 6-mm diameter discs were cut out of a matrix dried with 0.5 M trehalose:sucrose (50:50). Discs were weighed and, for each matrix type in turn, a total mass of 5–15 mg was loaded into Aluminium DSC pans (TA Instruments) and hermetically sealed. Samples were subjected to a temperature ramp from − 20 to 180 °C at a heating rate of 10 °C per minute. Measurements on all samples were performed in duplicates. Thermograms relating heat flow (W/g) to temperature (°C) were analysed using Trios software (TA Instruments) for identification of the glass transition (Tg) onset temperature.

For the measurement of enthalpic recovery, which manifests as an endothermic peak at the glass transition, modulated DSC (temperature modulation ± 0.50 °C every 60 s and ramp rate 3 °C/min from − 20 to 100 °C) was employed. The enthalpic recovery was estimated by linear peak integration in the thermograms plotted between nonreverse heat flow (W/g) and temperature (°C) using Universal Analysis software (TA Instruments).

### Protein recovery

A 10 mg/mL solution of lysozyme (Sigma-Aldrich) was made in 0.5 M trehalose sucrose. 25 µL was loaded into each matrix in triplicates. Protein was reconstituted from the matrices after desiccation overnight and recovery was quantified using EnzChek Lysozyme Assay Kit (ThermoFisher Scientific). Fluorescence measurements were performed in triplicates for each sample and protein recovery calculated by interpolation on a standard curve, using GraphPad Prism.

Protein loss was then calculated similarly to infectivity loss (above), i.e. log_10_-fold loss = log_10_(micrograms of protein dried on matrix) − log_10_(micrograms of protein recovered from matrix).

### Thermogravimetric analysis

Degradation temperatures of matrix constituents were measured by thermogravimetric analysis using a TGA Q500 (TA instruments). Samples loaded into a tared platinum pan just prior to measurement were subjected to a temperature ramp at 5 °C per minute from ambient to 550 °C in a flowing nitrogen atmosphere (100 mL/min). The gas was switched to air at 550 °C (100 mL/min) and heat was continued at the rate of 5 °C/min to 730 °C. Data was analysed using Universal Analysis software (TA Instruments).

### Polyvinyl alcohol (PVA) extraction and Fourier transform infrared spectroscopy

A 500 mm × 27 mm piece of glass fibre (S14) was dissolved in 100 mL of ultrapure water by stirring and heating at 90 °C for 48 h. The suspension was filtered through a 0.2 µm filter and the filtrate was freeze dried (Virtis AdVantage 2.0, SP Scientific) to a white amorphous material. This residue was subjected to single reflection attenuated total reflection (MIRAacle™, Pike Technologies) Fourier transform infrared spectroscopy ATR-FTIR (Tensor 37, Bruker) equipped with nitrogen-cooled mercury cadmium telluride detector.

To obtain a spectrum of the sample, an average of 64 interferograms was collected at a resolution of 4 cm^−1^ in the wavelength range from 4000 to 750 cm^−1^ and blank subtracted.

Spectra were analysed using OPUS 6.5 software (Bruker) and compared with RMIT University’s spectral library of organic compounds generated using Spectrum 10 software (Perkin Elmer). Fingerprint spectra shown in Fig. 5D were prepared using Prism 7.0 (GraphPad Software LLC).

### Dynamic light scattering (DLS)

The molecular weight of the PVA binder present on the glass fibre matrix (S14) was estimated by DLS. PVA was extracted and freeze-dried as described above. Solutions of this extracted sample and standards of known polymer size were prepared in water to the concentration of approximately 1.3 mg/mL and passed through 0.22 µm and 300 kDa molecular weight cut-off filters. The sample was then concentrated approximately five-fold from 4 mL to 0.75 mL using a 3 kDa MWCO filter (Vivaspin, GE). Zetasizer Nano ZS and DTS software (Malvern Instruments) was used for measurement of hydrodynamic diameter based on size distribution by volume. Independent duplicate preparations of all standards and samples were tested. Prism 7.0 (GraphPad Software LLC) was used to generate a standard curve plotted between measured hydrodynamic diameter and known average molecular weight (kDa) to interpolate size of the PVA in the extract.

### Nuclear magnetic resonance (NMR) spectroscopy

The degree of hydrolysis of the PVA binder present on the glass fibre matrix (S14) was estimated by ^1^H NMR measurement as the intensity of the peak attributable to the acetyl group present in non-hydrolysed PVA. A 5 mg/mL aqueous solution of PVA extracted from the glass fibre sample (S14) was prepared in deuterium oxide. Reference spectra for PVA with varying degrees of hydrolysis were obtained by mixing > 99% hydrolysed PVA and 80% hydrolysed PVA in appropriate proportions to produce standards containing c. 0%, 2%, 5%, 10% and 20% acetyl groups, again at 5 mg/mL in deuterium oxide. An AVIII 700 instrument (Bruker Biospin) was used to generate ^1^H 1D spectra (employing a quantitative 1D NOESY (Nuclear Overhauser Effect Spectroscopy) presaturation sequence with recovery delay d_1_ = 30 s).

### Application of PVA to matrices

Aqueous solutions of each type of PVA to be investigated were prepared at 10 mg/mL. Matrices were cut into approximately 100 mm^2^ pieces and loaded with until the matrices were saturated by the solution and air-dried overnight. The polyamide matrix (33100L) required surface modification by washing in 100% ethanol for 20 min and air drying before PVA could be loaded. Following PVA application, vaccines were dried on the matrices and thermostability assessed as described above.

### Study of physical characteristics of matrices

The physical characteristics of matrices were studied using NWSPs (Nonwoven Standard Procedures) prescribed by the EDANA, the international trade association for the nonwoven industry.

Areal density was measured as ratio of mass in grams and area in square metres according to EDANA NWSP 130.1R0 (15).

Matrix thickness was measured at an applied pressure of 0.5 kPa according to EDANA NWSP 120.1.R0 (15) using a thickness tester (ProGage, Thwing-Albert Instrument Company).

Fibre length was measured based on 60 individual fibres extracted from the matrix using a Leica MD G41 optical microscope in transmission mode and Image J software.

Minimum mean and maximum pore size were obtained using a POROLUX 100 Automated Capillary Flow Porometer using Galpore liquid of surface tension 15.6 mN∙m-1. A total of five measurements were taken per sample. Porosity (ɛ) was determined by the equation ɛ = (1 − ∅) × 100% where ∅ is the volume fraction measured as a ratio of bulk matrix density to bulk fibre density.

The surface tension of aqueous sugar solution on the matrix was measured on a Kruss K100 tensiometer using the Wilhelmy plate method in which the force exerted on a suspended plate when it touches the surface of a liquid is related to the surface tension and the contact angle according to the equation $\sigma = \frac{{\text{F}}}{{{\text{L}} \cdot \cos \theta }}$ where σ = surface tension of the liquid, F = measured force, L = wetted length, and Θ = contact angle.

Wettability was evaluated using the Washburn method, again on a Kruss K100 tensiometer. The rate of mass uptake when the porous substrate (matrix) comes in to contact with a liquid was used to determine the capillary constant of the substrate by applying the Washburn equation $\frac{{{\text{m}}^{2} }}{{\text{t}}} = \frac{{{\text{c}} \cdot \rho^{2} \cos \Theta }}{\eta }$, where m = mass, t = flow time, c = capillary constant of the nonwoven, ρ = density of the liquid, σ = surface tension of the liquid, Θ = contact angle, η = viscosity of the liquid. The capillary constant of the matrix was determined using n-hexane which has a contact angle of 0°.

### In-house production of glass-fibre-based matrix

A glass fibre matrix (Fig. 6C) was custom made by a wet-lay process using commercially available glass fibre and polyvinyl alcohol aiming for similar porosity, thickness, areal density and wetting behaviour as the glass fibre sample (S14).

Wet-lay processes are similar to paper manufacturing. Glass fibre with geometry similar to the fibres used in S14 was obtained (Johns Manville, type 475, diameter 4 µm). A slurry (suspension of fibres in water) was produced and agitated in a rig connected to water supply and drainage. Upon opening of the rig’s drainage outlet, the fibres were captured and retained on a fine metal mesh at the outlet, forming a nonwoven web. The fibrous mesh was blot dried, roller pressed and further dried at 110 °C for 15 min. 1 g/L PVA solution (> 99% hydrolysed, Mw 146,000–186,000 kDa, Sigma) was then applied to each side using a spray gun before drying at 110 °C for a further 15 min.
