# Publisher Correction: Characterisation of factors contributing to the performance of nonwoven fibrous matrices as substrates for adenovirus vectored vaccine stabilisation

**DOI:** 10.1038/s41598-021-02406-9

**Published:** 2021-11-29

**Authors:** Pawan Dulal, Robabeh Gharaei, Adam Berg, Adam A. Walters, Nicholas Hawkins, Tim D. W. Claridge, Katarzyna Kowal, Steven Neill, Adam J. Ritchie, Rebecca Ashfield, Adrian V. S. Hill, Giuseppe Tronci, Stephen J. Russell, Alexander D. Douglas

**Affiliations:** 1 Jenner Institute, University of Oxford, Wellcome Trust Centre for Human Genetics, Roosevelt Drive, Oxford, OX3 7BN UK; 2 Clothworkers’ Centre for Textile Materials Innovation for Healthcare, University of Leeds, Leeds, LS2 9JT UK; 3 Oxford Silk Group, ABRG, Department of Zoology, University of Oxford, Oxford, OX2 3RE UK; 4 Department of Chemistry, Chemistry Research Laboratory, University of Oxford, Mansfield Road, Oxford, OX1 3TA UK; 5 Nonwovens Innovation and Research Institute Ltd, 169 Meanwood Road, Leeds, LS7 1SR UK

Correction to: *Scientific Reports* 10.1038/s41598-021-00065-4, published online 22 October 2021

The original version of this Article contained an error in the spelling of the author Adam Berg which was incorrectly given as Adam J. Berg.

